# Skewed Production of IL-6 and TGFβ by Cultured Salivary Gland Epithelial Cells from Patients with Sjögren's Syndrome

**DOI:** 10.1371/journal.pone.0045689

**Published:** 2012-10-30

**Authors:** Takafumi Kawanami, Toshioki Sawaki, Tomoyuki Sakai, Miyuki Miki, Haruka Iwao, Akio Nakajima, Takuji Nakamura, Tomomi Sato, Yoshimasa Fujita, Masao Tanaka, Yasufumi Masaki, Toshihiro Fukushima, Yuko Hirose, Makoto Taniguchi, Naotoshi Sugimoto, Toshiro Okazaki, Hisanori Umehara

**Affiliations:** 1 Department of Hematology and Immunology, Kanazawa Medical University, Uchinada-machi, Kahoku-gun, Ishikawa, Japan; 2 Medical Research Institute, Kanazawa Medical University, Uchinada-machi, Kahoku-gun, Ishikawa, Japan; 3 Department of Physiology, Graduate School of Medical Science, Kanazawa University, Ishikawa, Japan; University of Bergen, Norway

## Abstract

**Objective:**

To determine the cytokine production profile of cultured salivary gland epithelial (SGE) cells obtained from patients with Sjögren's syndrome (SS).

**Methods:**

SGE cells obtained from 9 SS patients and 6 normal controls were cultured in the presence of exogenous IFNγ. Cell proliferation and apoptosis in response to IFNγ were determined by WST1 assay and by FACS analysis. The concentrations of IL-6 and TGFβ secreted into culture supernatants were analyzed by ELISA.

**Results:**

IFNγ did not significantly affect the proliferation or apoptosis of SGE cells. However, IL-6 concentrations were higher, and TGFβ concentrations were lower, in culture supernatants of SGE cells from SS patients than from normal controls.

**Conclusion:**

Cytokine production by SGE cells from SS patients showed a skewed balance compared with normal controls, with increased IL-6 and decreased TGFβ secretion. This imbalance may be critical in the regulation of Treg/Th17 cells and may foster a pathogenic milieu that may be causative and predictive in SS.

## Introduction

Sjögren's syndrome (SS) is a chronic autoimmune disease characterized by lymphocytic infiltration into the salivary and lacrimal glands [1], [2]. This chronic inflammation leads to destruction of the salivary glands and may ultimately result in salivary hypofunction. Although the mechanisms underlying this salivary gland destruction are not clearly understood, a better understanding of the precise molecular mechanisms may lead to the development of specific therapies for SS, similar to cytokine-targeted therapies in patients with rheumatoid arthritis (RA) [3], [4].

Cytokines are key molecules that mediate chronic autoimmune inflammatory reactions in the salivary glands of SS patients [5]. Proinflammatory cytokines, such as interferon (IFN) γ, interleukin (IL)-1β, IL-6, IL-10 and tumor necrosis factor (TNF) α, are produced by infiltrating lymphocytes and are involved in the maintenance of chronic inflammation [6]–[9]. In SS patients, these cytokines can induce the expression of HLA-DR, BAFF, costimulatory molecules such as CD80 and CD86, and/or chemokines in salivary gland epithelial (SGE) cells [10], [11]. In addition, we have reported that the production of IFNs can further perpetuate the homing and activation of lymphocytes and the apoptosis of glandular cells [12], [13]. In contrast, the absence of transforming growth factor (TGF) β has been reported to lead to systemic autoimmune diseases such as systemic lupus erythematosus (SLE) and SS in TGFβknockout mice [14], [15]. TGFβ promotes the differentiation of regulatory-T cells (Treg) [16] and, together with IL-6, plays a crucial role in the induction of Th17 cells [17], [18]. Taken together, these findings suggest that cytokine balance plays an important role in chronic inflammation of the salivary glands in SS patients [5]. Moreover, long-term exposure to pro-inflammatory cytokines such as IFNγ and TNFα can result in salivary epithelium dysfunction, leading to hyposalivation. We therefore evaluated cytokine expression profiles in salivary gland epithelial (SGE) cells from SS patients stimulated with IFNγ.

## Materials and Methods

### Patients and controls

We evaluated 15 patients at Kanazawa Medical University Hospital (Ishikawa, Japan) who were enrolled in the Sjögren's International Collaborative Clinical Alliance (SICCA) Registry; the complete details of this registry have been described [19]. In brief, SICCA is an ongoing longitudinal multisite observational study of a large and growing cohort of uniformly evaluated individuals from ethnically diverse populations, designed to develop standardized classification/diagnostic criteria for SS [20], [21]. Each participant in the SICCA cohort is assessed, systemically and extensively, for symptoms and signs related to SS. Of the 15 patients, nine (all women; mean age, 48±14 years) met both the 2002 American-European consensus group (AECG) and the SICCA criteria for SS [21], [22], whereas six (all women; mean age, 57±8 years) did not meet either set of criteria and had no objective findings indicative of SS (Table 1). All experimental protocols were approved by the independent ethics committee of Kanazawa Medical University, and all participants provided written informed consent.

**Table 1:** Profile of patients included in the study.

	Sex	Age	Diagnosis	Focus Score (/4 mm^2^)	ANA*	Anti SS-A	Anti SS-B
SS.1	F	64	SS	2.3	40	+	−
SS.2	F	67	SS	3.9	1280	−	−
SS.3	F	56	SS	1.8	160	+	+
SS.4	F	44	SS	2.8	320	+	+
SS.5	F	28	SS	3.2	160	+	−
SS.6	F	32	SS	2.4	80	+	+
SS.7	F	58	SS	2.7	160	+	−
SS.8	F	36	SS	2.9	160	+	+
SS.9	F	52	SS	1.2	−	+	−
No.1	F	67	non-SS	0	320	−	−
No.2	F	67	non-SS	0	−	−	−
No.3	F	51	non-SS	0	−	−	−
No.4	F	57	non-SS	0.33	−	−	−
No.5	F	46	non-SS	0	−	−	−
No.6	F	58	non-SS	0	640	−	−

Nine patients (all women; mean age, 48±14 years) met both the 2002 American-European consensus group (AECG) criteria and the SICCA criteria for Sjögren's syndrome (SS), whereas the other six (all women; mean age, 57±8 years) did not (No).

* Titers of anti-nuclear antibody (ANA).

Labial minor salivary gland (MSG) biopsies were taken from each patient for diagnostic evaluation of SS, with SG tissue samples processed for further culture of primary epithelial cells. None of these participants had taken any immune suppressants or steroids.

### Cell lines and primary cultures of SGE cells from MSGs

Human airway epithelial cells (HBTEC) and human umbilical vein endothelial cells (HUVEC) were obtained from Kurabo Co. Ltd., Osaka Japan. Epithelial cells obtained from the MSGs were cultured immediately after biopsy, as described [13]. In brief, each tissue sample was rinsed with cold sterile phosphate-buffered saline (PBS) containing 100 U/ml penicillin and 100 µg/ml streptomycin and minced into small pieces of approximately 1–2 mm^3^. One tissue sample from each subject was placed in a well of a collagen type 1-coated 12-well plate (Iwaki, Tokyo, Japan) and cultured in keratinocyte serum-free medium (SFM; Invitrogen Corp., Carlsbad, CA) containing 0.4 µg/ml hydrocortisone and 25 µg/ml bovine pituitary extract (Sigma Chemical Co., St. Louis, MO). The epithelial cell outgrowth of each explant was assessed after 1–2 weeks. Upon attaining confluence, the monolayer cells were subcultured. Cells were rinsed twice with PBS and detached from the substrate by incubation with 0.05% trypsin (Invitrogen) for no longer than 10 min, with cell detachment monitored by light microscopy. Trypsin was inactivated by adding an equal volume of Dulbecco's modified Eagle's medium (DMEM; Invitrogen) supplemented with 10% fetal calf serum (FCS). The detached cells were centrifuged at 1500 rpm for 5 min, washed once with PBS, resuspended in culture medium, and reseeded, at a concentration of 8×10^4^ cells per well, in a fresh collagen type I-coated 6-well plate. Fibroblasts were routinely removed from the cultures by treating the cells with 0.02% ethylenediaminetetraacetic acid (EDTA; Invitrogen).

### Immunocytochemistry

The SGE cells were harvested, washed once with PBS, and allowed to adhere to a glass slide in a monolayer. The cells were air dried for 30 min, fixed in 4% paraformaldehyde (PFA) acetone at 4°C for 30 sec, washed 3 times with purified water, incubated in Tris-buffered saline (TBS) for 5 min, and blocked with bovine serum albumin at room temperature for 5 min. After washing, the cells were incubated overnight at 4°C in a moist chamber with primary antibodies against epithelial membrane antigen (EMA, clone E29), cytokeratin 8 (35βH11), and cytokeratin 18 (DC10; Dako, Kyoto, Japan), each at a concentration of 1 µg/ml. Antigen-antibody complexes were detected using a labeled polymer conjugated with alkaline phosphatase (Envision/AP kit, Dako), according to the manufacturer's instructions, visualized by treatment for 10 min with the New Fuchsin chromogen-substrate solution and counterstained with Mayer's hematoxylin. Control slides were incubated with isotype-matched antibodies in TBS in place of the primary antibody; these invariably yielded negative results (data not shown).

### Proliferation assay and detection of cell apoptosis

The proliferative responses of SGE cells to IFNγ (R&D Systems) were determined using a WST-1 cell counting kit, and cell apoptosis was determined by flow cytometry as described [23]. Briefly, SGE cells were cultured in collagen type I-coated plates in the presence of IFNγ at 37°C for the indicated times. Ethanol-fixed cell suspensions were centrifuged, followed by the addition of 50 µl RNase solution and 450 µl propidium iodide (final concentration 50 mg/ml). The cells were washed twice and subsequently analyzed by flow cytometry (BD Biosciences, Palo Alto, CA, USA).

### Quantification of cytokines in the culture supernatants by ELISA

The concentrations of IL-6 and TGFβ1 secreted into culture supernatants were quantified using ELISA test kits for IL-6 (Immunotech, Marseille, France) and TGFβ1 (R&D Systems, Minneapolis, MN), according to the manufacturers' instructions.

### Statistical analysis

Median cytokine concentrations, calculated to minimize the effects of extreme outliers, were compared using non-parametric Mann-Whitney tests. A p value<0.05 was considered statistically significant.

## Results

### Primary cultures of human SGE cells from MSGs

**Figure 1:**
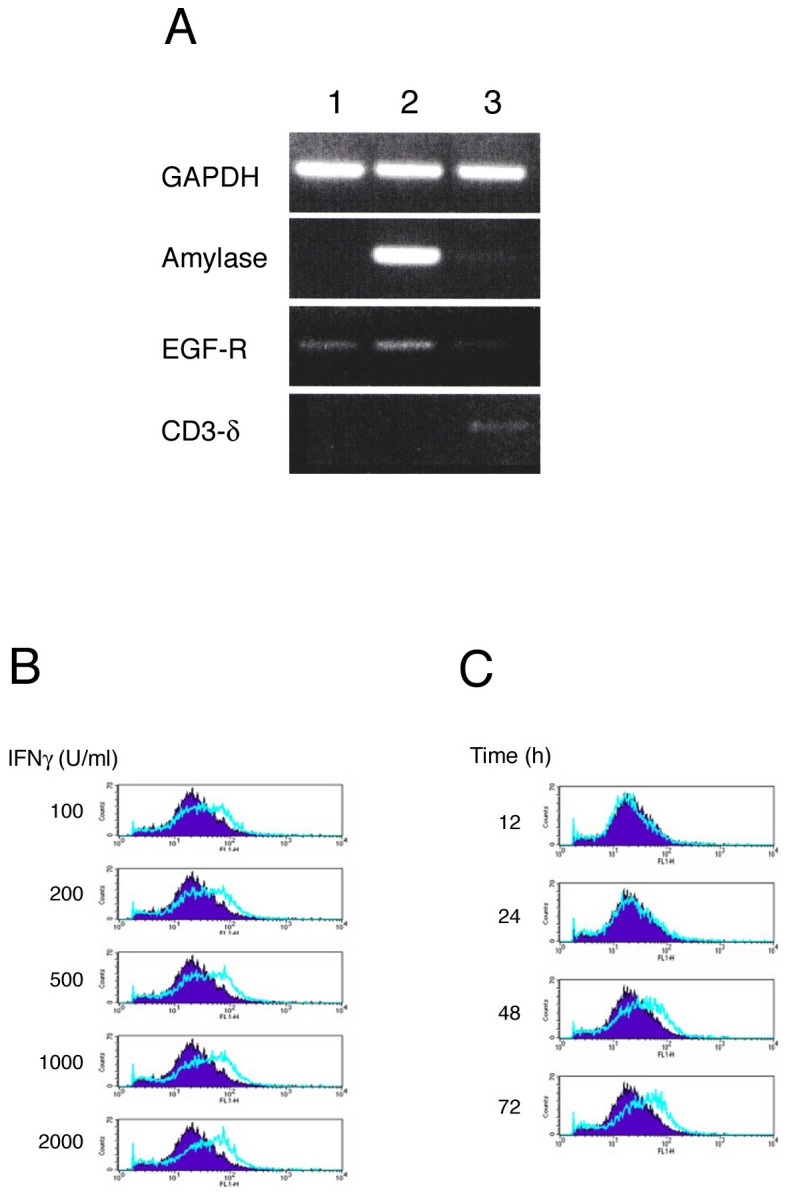
(A) Expression of mRNA in salivary gland epithelial cells. Salivary gland epithelial cells were isolated from SS patients and cultured. Total RNA was isolated from these cells, and EGF-R, α amylase-1, and CD3δ mRNAs were assayed by RT-PCR, as described in the Materials and Methods section. Lane 1: salivary gland epithelial cells, Lane 2: labial salivary gland of the same patient, Lane 3: normal lymph node as a control for CD3δ. **(B and C) Effects of IFNγ on human SGE cells.** SGE cells were incubated with various concentration of IFNγ for 48 hours (B) or with 1000 U/ml of IFNγ for the indicated times (C), and the surface expression of CD40 was examined by FACS analysis.

To establish primary cultures of human SGE cells, MSG biopsy samples were washed with PBS, cut into pieces of approximately 1–2 mm^3^, and explanted onto collagen type 1-coated plastic plates. Within a few weeks, the epithelial cells gradually grew out from the explants. Most of these cells were cuboidal, round, or spindle shaped. RT-PCR showed that these cells expressed EGF-R, but not α-amylase 1 or CD3δ, mRNA (Figure 1A). Immunohistochemical analysis showed that these cells were positive for EMA and cytokeratins-8 and -18 (data not shown), indicating that these cells were primarily ductal epithelial cells, with small amounts of acinar or lymphocytic components. These SGE cells could be maintained in culture medium for at least a few months.

### Effects of IFNγ on activation of SGE cells

Previous studies have shown that the concentrations of cytokines such as IFNγ, IL-1β, IL-6, IL-10 and TNFα are increased in SS patients, and that long-term exposure to pro-inflammatory cytokines such as IFNγ and TNFα can lead to dysfunction of the salivary epithelium. To assess the effects of IFNγ on SGE cell activation, SGE cells were incubated with various concentration of IFNγ for 48 hours (Figure 1B) or with 1000 U/ml of IFNγ for the indicated times (Figure 1C), and the expression of CD40 was examined by FACS analysis. We found that CD40 expression on SGE cells was increased in a dose- and time-dependent manner (Figure 1B and C).

### Effects of IFNγ on the proliferation and apoptosis of SGE cells

**Figure 2:**
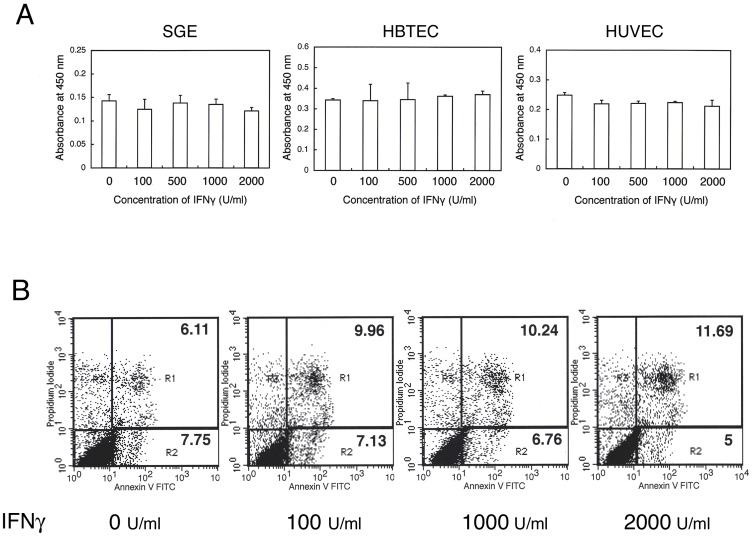
Effects of IFNγ on the proliferation and apoptosis of SGE cells. (A) SGE cells, human airway epithelial cells (HBTEC) and human umbilical vein endothelial cells (HUVEC)were incubated with the indicated concentration of IFNγ, and proliferative responses were assessed at 48 h. Each bar shows mean + SD. IFNγ did not significantly affect the proliferation of any of these cells. The results shown are representative of three independent experiments. (B) SGE cells were incubated with the indicated concentration of IFNγ, and apoptosis was determined at 12 h by flow cytometry. Numbers in R1 and R2 indicate early and late apoptosis, respectively.

To test the effects of IFNγ on the proliferation of SGE cells, these cells, as well as human airway epithelial cells (HBTEC) and human umbilical vein endothelial cells (HUVEC), were incubated in the presence of the indicated concentrations of IFNγ for 48 h. We found that IFNγ had no effects on the proliferation these three cell types (Figure 2A). To assess the effects of IFNγ on SGE cell apoptosis, these cells were incubated with the indicated concentrations of IFNγ for 12 h. We observed that IFNγ did not significantly affect the early (R1) or late (R2) apoptosis of SGE cells (Figure 2B).

### Production of IL-6 and TGFβ by IFNγ stimulated SGE cells

**Figure 3:**
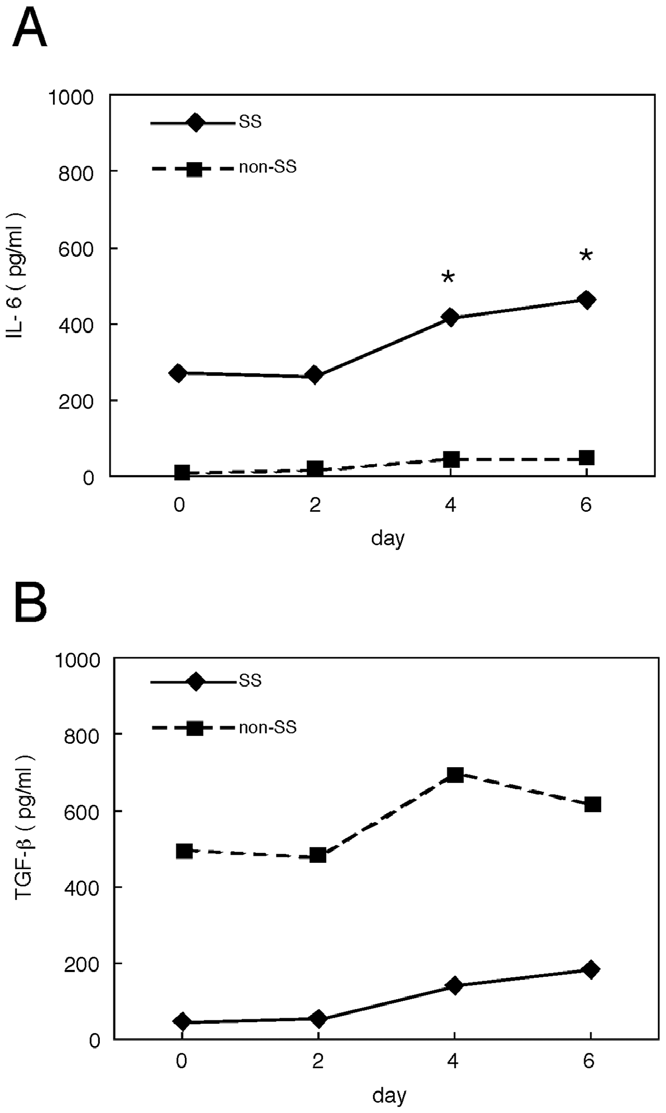
Quantification of cytokines secreted into the culture supernatants of SGE cells. Confluent SGE cells obtained from 9 SS patients and 6 normal controls (non-SS) were incubated in the presence of IFNγ (1000 U/ml), and the culture supernatants were collected on days 0, 2, 4, and 6. Median concentrations (pg/ml) of IL-6 (A) and TGFβ (B) in the supernatants were determined by ELISA and compared by non-parametric Mann-Whitney tests (*, p<0.05).

TGFβ induces Foxp3 in naïve T cells, resulting in their differentiation to regulatory T cells (Treg). In contrast, IL-6 switches T cell differentiation from a Treg to a Th17 pathway. We therefore incubated confluent SGE cells with IFNγ (1000 U/ml), and assayed the concentrations of IL-6 and TGFβ secreted into culture supernatants on days 0, 2, 4 and 6. We found that IL-6 concentrations on days 4 and 6 were significantly higher (Figure 3A), and TGFβ concentrations on days 2 and 4 were significantly lower (Figure 3B), in the supernatants of cells from SS patients than from controls.

## Discussion

Although the production of cytokines in the salivary glands of SS patients has been evaluated [6]–[9], [12], [13], the most important cytokines involved in the pathogenesis of SS have not yet been identified. Since it is difficult to distinguish between cause and effect relationships of cytokine production, due to their complex interactions and the presence of various cells in tissues, we attempted to minimize this problem by using cultured SGE cells. Although previous studies have been hampered by the lack of a suitable in vitro culture system for SGE cells, our culture system, using a non-serum-containing medium, enabled us to examine cellular functions including cytokine production and to maintain human SGE cells as ductal epithelial cells for at least a few months.

Because the salivary glands of SS patients contain T cells that express IFNγ and Stat1 mRNA [24], with these T cells being predominantly Th1 cells [25], the proinflammatory cytokine IFNγ has been considered a principle mediator of inflammation in SS patients, similar to TNFα in patients with RA [3]. Local IFNγ production in the salivary glands may perpetuate inflammation by inducing SGE cells to express HLA-DR, co-stimulatory molecules, cytokines and chemokines, leading to secretory gland dysfunction [2]. We therefore comprehensively examined the effects of IFNγ on the functions of SGE cells obtained from SS patients. We found that IFNγ activated SGE cells, leading to the increased expression of CD40, but did not significantly affect the proliferation or apoptosis of SGE cells.

Next we examined cytokine production by IFNγ-stimulated SGE cells obtained from SS patients. Although the differences were not significant, IFNγ induced a skewed expression of mRNAs encoding several cytokines, including IL-6, TNFα, TGFα and TGFβ, in SGE cells from both SS patients and normal controls (data not shown). We hypothesized that TGFβand IL-6 may play key roles in the pathogenesis of SS by affecting the balance between Treg and Th17 cells. TGFβ can have pro- or anti-inflammatory effects, depending on the context; i.e., TGFβ promotes the differentiation of naive T cells to Treg cells in the presence of IL-2, while inducing Th17 cells in the presence of IL-6 [16], [26], [27].

IL-6 is another pleiotropic cytokine that regulates immune responses, hematopoiesis and bone metabolism [28]. IL-6 overproduction has been found to be involved in the pathogenesis of several human autoimmune diseases, including RA and Castleman's disease [29]. Recently, IL-6 was shown to play an important role in T helper differentiation [26]. Stimulation of cultured CD4 T cells with IL-6 and TGFβ potently induced Th17 differentiation. IL-17 has been reported to be involved in the chronic inflammatory processes that occur in many autoimmune diseases, including SS [27], [30]. Whereas TGFβ was found to induce naive CD4 T cells to differentiate into Foxp3+ Treg cells, this Treg induction was potently inhibited by IL-6. Rather, IL-6 was found to promote their differentiation into inflammatory Th17 cells, providing further evidence that IL-6 is critical in the regulation of the Treg/Th17 balance [26].

To identify the cytokines that play major roles in the development of salivary gland lesions in SS, we assayed the concentrations of IL-6 and TGFβ secreted into the culture supernatants of IFNγ-stimulated SGE cells. We found that IL-6 concentrations were higher in the supernatants of cells from SS patients than from normal controls, suggesting that IL-6 may be important in maintaining chronic inflammation in SS. These findings are consistent with results showing that IL-6 is highly expressed in SGE, with high focal scores [31]. Moreover, increased IL-6 production may contribute to the presence of abundant IL-17-bearing cells in the salivary glands of SS patients [30].

In contrast, we found that the secretion of TGFβ was lower in supernatants of SGE cells from SS patients than from controls. Since TGFβ has been linked to the generation of Treg cells, it is likely that Th1 cell differentiation and IFNγ production are not suppressed in the salivary glands of SS patients during the initial stages of inflammation.

Our findings suggested that the skewed production of IL-6 and TGFβ in the salivary glands of SS patients may shift the milieu to one more favorable for the propagation of Th17 cells, with correspondingly fewer Foxp3+ Treg cells, and may foster a pathogenic milieu that may be causative and predictive of infiltrative injury in SS patients.

Although the mechanism underlying this cytokine imbalance in SGE cells of SS patients, including reduced TGFβ and increased IL-6 production is currently unknown, modulation of this imbalance may help control the chronic inflammation of the salivary glands occurring in SS patients.
